# Supplementary material for: Vaccine hesitancy among paediatric nurses: Prevalence and associated factors
Source: PLoS One. 2021 May 19;16(5):e0251735. doi: 10.1371/journal.pone.0251735 (PMC8133484; doi:10.1371/journal.pone.0251735)
Supplement: S1 Appendix — (PDF) [file pone.0251735.s001.pdf]

S1-Appendix

The questionnaire of our study in Barcelona was based on three previous validated questionnaires:

1. Questionnaire from the study of Posfay-Barbe 2005

Posfay-Barbe KM, Heininger U, Aebi C, Desgrandchamps D, Vaudaux B, Siegrist CA. How do physicians immunize their own children? Differences among pediatricians and non pediatricians. Pediatrics. 2005 Nov;116(5):e623-33.

How do physicians immunize their own children?

1. Do you have children ?
   - No ❑ Please go directly to question 7!
   - Yes - < 5 years old ❑
     - 5-15 years old ❑ *(several answers possible)*
     - > 15 years old ❑

2. Which vaccine(s) would you give to your **own** children? *(several answers possible)*
   - B.C.G. ❑
   - Diphtheria ❑
   - Tetanos ❑
   - Pertussis ❑
   - Polio ❑
   - Hib ❑
   - Measles ❑
   - Rubella ❑
   - Mumps ❑
   - Hepatitis B ❑
   - Hepatitis A ❑
   - Pneumococcal ❑
   - Meningococcal C ❑
   - Varicella ❑
   - Tick-born encephalitis ❑
   - Flu ❑
   - Other vaccine(s) 1. ………………………… 2. …………………………

3. Are there 2004 recommended vaccines that you **didn't want** give to your **own children** ? *(several answers possible)*
   - I gave all vaccines that were available at that time ❑
   - I didn't give any recommended vaccines ❑
   - I didn't give tetanos ❑ because: ……………
   - I didn't give diphtheria ❑ because: ……………
   - I didn't give pertussis ❑ because: ……………
   - I didn't give combined DTP/DTaP ❑ because: ……………
   - I didn't give Hib ❑ because: ……………
   - I didn't give polio ❑ because: ……………
   - I didn't give measles ❑ because: ……………
   - I didn't give rubella ❑ because: ……………
   - I didn't give mumps ❑ because: ……………
   - I didn't give MMR ❑ because: ……………
   - I didn't give hepatitis B ❑ because: ……………

4. Did you decide to postpone the first dose of **DTP/DTaP** of **your own children?** *(several answers possible if different for each child)*
   - no, immunization between approximately 2 and 6 months ❑
   - yes, immunization between approximately 6 and 12 months ❑
   - yes, immunization between approximately 12 and 14 months ❑
   - yes, immunization > 24 months ❑
   - not immunized ❑

Fig 1. InfoVac Web-based questionnaire.

acteristics. In general, the time since qualification or region of practice had no statistically significant effect on vaccine use. Nine hundred fifteen (90%) physicians had ≥1 children (24% younger than 5 years of age, 50% between 5 and 15 years, and 52% older than 15 years of age). Women were more likely to be pediatricians, and pediatricians worked more often in private practice than nonpediatricians. Pediatricians were also less likely to belong to a self-reported alternative medicine association (3.1% vs 7%; P =

www.pediatrics.org/cgi/doi/10.1542/peds.2005-0885 e625
Downloaded from pediatrics.aappublications.org at Harvard University on June 12, 2015

5. Did you decide to postpone the first dose of **measles/MMR** of **your own children?** *(several answers possible if different for each child)*

- no, immunization between 12 and 24 months ❑
- yes, immunization between 2 and 5 years ❑
- yes, immunization between 5 and 10 years ❑
- yes, immunization between 10 and 15 years ❑
- yes, immunization > 15 years ❑
- not immunized ❑

6. Do you think that your **own children** have been immunized **differently** then children/patients in your own practice ? (*several answers possible*)

- yes, my children received more vaccines ❑
- yes, my children received less vaccines ❑
- yes, my children have been immunized earlier ❑
- yes, my children have been immunized later ❑
- no, no difference ❑

7. If you were a "new parent" in 2004, **which vaccines** would you give to **your own children**? *(several answers possible)*

- B.C.G. ❑
- Diphtheria ❑
- Tetanos ❑
- Pertussis ❑
- Polio ❑
- Hib ❑
- Measles ❑
- Rubella ❑
- Mumps ❑
- Hepatitis B ❑
- Hepatitis A ❑
- Pneumococcal ❑
- Meningococcal C ❑
- Varicella ❑
- Tick-born encephalitis ❑
- Flu ❑
- Other vaccine(s) 1. ....2. ....

8. If you were a "new parent" in 2004, **which combination vaccine** would you give to **your own children?** *(several answers possible)*

- DTaP ❑
- DTaP-IPV ❑
- DTaP-Hib ❑
- DTaP-IPV/Hib ❑
- Hexavalent ❑
- MMR ❑
- Hepatitis A/B ❑
- no combination vaccine ❑

9. If you were a "new parent" in 2004, **at what age** would you give **the first dose** of **DTaP** to **your own children** ?

- 2-4 months ❑
- 5-6 months ❑
- 7-12 months ❑
- > 12 months ❑
- > 24 months ❑
- not vaccinated ❑

Fig 1. Continued.

Downloaded from pediatrics.aappublications.org at Harvard University on June 12, 2015

10. If you were a "new parent" in 2004, **at what age** would you give the first **measles or MMR vaccine** to **your own children** ?

- < 2 years ❑
- 2-5 years ❑
- 6-10 years ❑
- 11-15 years ❑
- > 15 years ❑
- not vaccinated ❑

11. If you were a "new parent" in 2004, are there any combination vaccines **recommended** by the Swiss vaccine schedule that you wouldn't give to your **own children** ? *(several answers possible)*

- I would give all recommended vaccines ❑
- I wouldn't give any recommended vaccines ❑
- no diphtheria vaccine ❑ because: ....
- no tetanus vaccine ❑ because: ....
- no pertussis vaccine ❑ because: ....
- no DTPa vaccine ❑ because: ....
- no polio vaccine ❑ because: ....
- no Hib vaccine ❑ because: ....
- no DTPa-IPV/Hib vaccine ❑ because: ....
- no hexavalent vaccine ❑ because: ....
- no measles vaccine ❑ because: ....
- no rubella vaccine ❑ because: ....
- no mumps vaccine ❑ because: ....
- no MMR vaccine ❑ because: ....
- no hepatitis B vaccine ❑ because: ....

To help us identify some factors that might influence the way physicians immunize their own children, please answer to the following questions :

You are : *(several answers possible)*

- a man ❑
- a woman ❑
- a pediatrician ❑
- a general practitioner ❑
- an internist ❑
- another specialist ❑

You finished your medical school :

- ≥ 2000 ❑
- between 1990-1999 ❑
- between 1980-1989 ❑
- between 1970-1979 ❑
- between 1960-1969 ❑
- < 1960 ❑

You live in ____________ (list of cantons)
You work in ___________ (list of cantons)

You work : *(several answers possible)*

- in private practice ❑
- at a hospital ❑
- in public administration ❑
- in school medecine ❑
- in the pharmaceutical industry ❑
- other professional area : ....

Fig 1. Continued.

Downloaded from pediatrics.aappublications.org at Harvard University on June 12, 2015

Of which medical association are you a member : *(several answers possible)*

- SSP/SGP (Pediatric) ❑
- Forum für Praxispädiatrie (Pediatric) ❑
- SSMG/SGAM (general medecine) ❑
- CMPR/KHM (primary care medecine) ❑
- SSMI/SGIM(internal medecine) ❑
- SSI/SGInf (infectiology) ❑
- Alternative medecine association ❑

Where do you find information concerning immunization in your daily practice (1 to 5, 1 being the most useful) :

- documents of the Swiss Federal Office of Public Health ❑
- documents of Forum für Praxispädiatrie ❑
- documents prepared by the pharmaceutical industry ❑
- documents distributed by Infovac ❑
- other documents ❑
  (such as : ……………………………….)

We thank you for your participation and look forward to sharing the results with you.

Your InfoVac experts

2. Questionnaire from the study of Buxton 2013

Buxton JA, McIntyre CC, Tu AW, Eadie BD, Remple VP, Halperin B, Pielak KL. Who knows more about immunization?: Survey of public health nurses and physicians. Can Fam Physician. 2013 Nov;59(11):e514-21.

The questionnaire and validation study:

Pielak KL, McIntyre CC, Tu AW, Remple VP, Halperin B, Buxton JA. Identifying attitudes, beliefs and reported practices of nurses and doctors as immunization providers. J Adv Nurs. 2010 Jul;66(7):1602-11.

---

3. Questionnaire from the study of Salmon 2004

Salmon DA, Moulton LH, Omer SB, Chace LM, Klassen A, Talebian P, Halsey NA. Knowledge, attitudes, and beliefs of school nurses and personnel and associations with nonmedical immunization exemptions. Pediatrics. 2004 Jun;113(6):e552-9.
