# Supplementary material for: Vaccine hesitancy among paediatric nurses: Prevalence and associated factors
Source: PLoS One. 2021 May 19;16(5):e0251735. doi: 10.1371/journal.pone.0251735 (PMC8133484; doi:10.1371/journal.pone.0251735)
Supplement: S2 Appendix — (PDF) [file pone.0251735.s002.pdf]

S2 Appendix. Questionnaire 1 – Spanish and English version

CSB Consorci Sanitari de Barcelona

Agència de Salut Pública

ENCUESTA A PROFESIONALES SANITARIOS SOBRE VACUNACIÓN SISTEMÁTICA

Esta encuesta forma parte de un estudio sobre vacunación infantil de la Agencia de Salud Pública de Barcelona dirigido a personal sanitario de Centros de Atención Primaria de la ciudad. La encuesta es de participación voluntaria y anónima, y sus respuestas serán tratadas confidencialmente. Gracias por su colaboración.

Datos generales

Usted es:

☐ Mujer

☐ Home

Edad: |__|__| años

Señale la profesión que ejerce:

☐ Medicina

☐ Enfermería

Indique los años aproximados que hace que ejerce esta profesión: |__|__| años

¿Tiene hijos/as?

☐ No

☐ Sí

1

60905

Preguntas sobre vacunación

1 - **Si usted hoy tuviera un hijo o hija, ¿qué vacunas del calendario sistemático vigente le administraría? Por favor, indique si se las administraría en las franjas de edad que marca el calendario (SÍ), si se las administraría más tarde (TARDE), si tendría dudas (DUDAS) o si no se las administraría (NO):**

	SÍ	TARDE	DUDAS	NO
Vacuna de la difteria	○	○	○	○
Vacuna del tétanos	○	○	○	○
Vacuna de la tos ferina	○	○	○	○
Vacuna de la poliomielitis	○	○	○	○
Vacuna del *H. influenzae* b	○	○	○	○
Vacuna de la hepatitis B	○	○	○	○
Vacuna del meningococo C	○	○	○	○
Vacuna de la hepatitis A	○	○	○	○
Vacuna del sarampión	○	○	○	○
Vacuna de la rubéola	○	○	○	○
Vacuna de la parotiditis	○	○	○	○
Vacuna del VPH *(chicas)*	○	○	○	○
Vacuna de la varicela	○	○	○	○
Vacuna del neumococo	○	○	○	○

Y ¿qué vacunas combinadas le administraría?

	SÍ	TARDE	DUDAS	NO
Vacuna dTpa<br>(difteria, tétanos, tos ferina acelular)	○	○	○	○
Vacuna pentavalente<br>(dTpa, polio, *H. influenzae* b)	○	○	○	○
Vacuna hexavalente<br>(dTpa, polio, *H. influenzae* b, hepatitis B)	○	○	○	○
Vacuna triple vírica<br>(sarampión, rubéola, parotiditis)	○	○	○	○

2 - ¿Qué PROBABILIDAD considera usted que tiene de contagiarse de las siguientes enfermedades un niño o niña NO vacunado/a ni inmunizado/a? Considere un periodo de 10 años en el contexto español actual.

-

	IMPOSIBLE	POCO PROBABLE	NI PROBABLE NI IMPROBABLE	PROBABLE	MUY PROBABLE	NS/NC
Difteria	○	○	○	○	○	○
Tétanos	○	○	○	○	○	○
Tos ferina	○	○	○	○	○	○
Poliomielitis	○	○	○	○	○	○
Enf. por *H. influenzae* b	○	○	○	○	○	○
Hepatitis B	○	○	○	○	○	○
Enf. por meningococo C	○	○	○	○	○	○
Hepatitis A	○	○	○	○	○	○
Sarampión	○	○	○	○	○	○
Rubéola	○	○	○	○	○	○
Parotiditis	○	○	○	○	○	○
Infección por VPH *(chicas)*	○	○	○	○	○	○
Varicela	○	○	○	○	○	○
Enf. por neumococo	○	○	○	○	○	○

3 - Esta pregunta trata sobre la GRAVEDAD de las enfermedades vacunables. ¿Cuán grave considera usted que es que un/a niño/a de 8 años no inmunizado se contagie de las siguientes enfermedades?

	NADA GRAVE	POCO GRAVE	MEDIANAMENTE GRAVE	GRAVE	MUY GRAVE	NS/NC
Difteria	○	○	○	○	○	○
Tétanos	○	○	○	○	○	○
Tos ferina	○	○	○	○	○	○
Poliomielitis	○	○	○	○	○	○
Enf. por *H. influenzae* b	○	○	○	○	○	○
Hepatitis B	○	○	○	○	○	○
Enf. por meningococo C	○	○	○	○	○	○
Hepatitis A	○	○	○	○	○	○
Sarampión	○	○	○	○	○	○
Rubéola	○	○	○	○	○	○
Parotiditis	○	○	○	○	○	○
Varicela	○	○	○	○	○	○
Enf. por neumococo	○	○	○	○	○	○

4 - **Esta pregunta trata sobre la SEGURIDAD de las vacunas. ¿Cuán seguras cree usted que son las vacunas siguientes?**

	PELIGROSA	POCO SEGURA	SEGURA	MUY SEGURA	TOTALMENTE SEGURA	NS/NC
Vacuna de la difteria	○	○	○	○	○	○
Vacuna del tétanos	○	○	○	○	○	○
Vacuna de la tos ferina	○	○	○	○	○	○
Vacuna de la poliomielitis	○	○	○	○	○	○
Vacuna del *H. influenzae* b	○	○	○	○	○	○
Vacuna de la hepatitis B	○	○	○	○	○	○
Vacuna del meningococo C	○	○	○	○	○	○
Vacuna de la hepatitis A	○	○	○	○	○	○
Vacuna del sarampión	○	○	○	○	○	○
Vacuna de la rubéola	○	○	○	○	○	○
Vacuna de la parotiditis	○	○	○	○	○	○
Vacuna del VPH *(chicas)*	○	○	○	○	○	○
Vacuna de la varicela	○	○	○	○	○	○
Vacuna del neumococo	○	○	○	○	○	○

5 - **¿Qué PROTECCIÓN cree que ofrecen las vacunas siguientes? Indique en qué grado considera que estas vacunas evitan que los niños y niñas se contagien de las enfermedades para las cuales están diseñadas:**

	NADA PROTECTORA	POCO PROTECTORA	MEDIANAMENTE PROTECTORA	PROTECTORA	TOTALMENTE PROTECTORA	NS/NC
Vacuna de la difteria	○	○	○	○	○	○
Vacuna del tétanos	○	○	○	○	○	○
Vacuna de la tos ferina	○	○	○	○	○	○
Vacuna de la poliomielitis	○	○	○	○	○	○
Vacuna del *H. influenzae* b	○	○	○	○	○	○
Vacuna de la hepatitis B	○	○	○	○	○	○
Vacuna del meningococo C	○	○	○	○	○	○
Vacuna de la hepatitis A	○	○	○	○	○	○
Vacuna del sarampión	○	○	○	○	○	○
Vacuna de la rubéola	○	○	○	○	○	○
Vacuna de la parotiditis	○	○	○	○	○	○
Vacuna del VPH *(chicas)*	○	○	○	○	○	○
Vacuna de la varicela	○	○	○	○	○	○
Vacuna del neumococo	○	○	○	○	○	○

6 - **Esta pregunta trata sobre el BENEFICIO que supone la vacunación de un/a niño/a para diferentes personas o grupos. ¿Cuánto cree usted que se benefician las siguientes personas o grupos cuando un/a niño/a recibe las vacunas del calendario vacunal vigente?**

	NADA	POCO	INTERMEDIO	BASTANTE	MUCHO	NS/NC
El/la niño/a se beneficia	○	○	○	○	○	○
La comunidad se beneficia (familia, amigos, barrio...)	○	○	○	○	○	○
El personal sanitario se beneficia	○	○	○	○	○	○
El gobierno/administración se beneficia	○	○	○	○	○	○
Las compañías farmacéuticas se benefician	○	○	○	○	○	○

7 - **A continuación, se alistan diversas afirmaciones sobre las vacunas infantiles. Indique su grado de acuerdo con ellas:**

	NADA DE ACUERDO	POCO DE ACUERDO	NI DE ACUERDO NI EN DES-ACUERDO	DE ACUERDO	MUY DE ACUERDO	NS/NC
Los niños y niñas sólo se deberían vacunar de enfermedades graves.	○	○	○	○	○	○
Los niños y niñas reciben más vacunas de las que necesitan.	○	○	○	○	○	○
Me preocupa que el sistema inmunitario de los niños y niñas pueda debilitarse debido a recibir demasiadas vacunas.	○	○	○	○	○	○
Soy más propenso/a a confiar en las vacunas que hace tiempo que se usan, comparado con las más nuevas.	○	○	○	○	○	○
Las vacunas son una de las medidas sanitarias más seguras que existen.	○	○	○	○	○	○
Gracias a la investigación, las vacunas son cada vez mejores y más eficaces.	○	○	○	○	○	○

5

60905

	NADA DE ACUERDO	POCO DE ACUERDO	NI DE ACUERDO NI EN DES-ACUERDO	DE ACUERDO	MUY DE ACUERDO	NS/NC
Las vacunas fortalecen el sistema inmunitario.	○	○	○	○	○	○
Es mejor que los niños y niñas desarrollen inmunidad poniéndose enfermos que vacunándose.	○	○	○	○	○	○
Continuar vacunando contra la polio en España es adecuado, aunque haya sido eliminada del país.	○	○	○	○	○	○
Creo que al menos alguna de las vacunas del calendario vigente se administra demasiado temprano.	○	○	○	○	○	○
Creo que las vacunas del calendario vacunal están influidas por intereses ilegítimos del gobierno.	○	○	○	○	○	○
Creo que las vacunas del calendario vacunal están influidas por intereses ilegítimos de la industria farmacéutica.	○	○	○	○	○	○
La vacuna triple vírica puede ser una causa de autismo.	○	○	○	○	○	○
La vacuna de la varicela puede ser una causa de varicela atenuada.	○	○	○	○	○	○
Al menos una vacuna del calendario vacunal infantil contiene timerosal.	○	○	○	○	○	○
La cantidad de timerosal empleada en vacunas puede ser una causa de neurotoxicidad.	○	○	○	○	○	○
Al menos una vacuna del calendario vacunal infantil contiene aluminio.	○	○	○	○	○	○
La cantidad de aluminio empleada en vacunas puede ser una causa de neurotoxicidad.	○	○	○	○	○	○
La alergia al huevo es una contraindicación para la triple vírica.	○	○	○	○	○	○
Las personas de mi entorno cercano están a favor de la vacunación.	○	○	○	○	○	○

8 - En su práctica profesional, ¿ha atendido usted a familias con dudas o contrarios a alguna vacuna?

☐ SI

☐ No *(pase a la pregunta 10)*

9 - A continuación, se alistan diversos motivos descritos como fuente de duda o rechazo vacunal de las familias. Valórelos según la IMPORTANCIA que percibe usted que tienen para las familias dudosas/contrarias a las vacunas que atiende en su día a día:

	NADA IMPORTANTE	POCO IMPORTANTE	MEDIANAMENTE IMPORTANTE	BASTANTE IMPORTANTE	MUY IMPORTANTE	NS/NC
Dudas sobre la seguridad de las vacunas	○	○	○	○	○	○
Dudas sobre la eficacia de les vacunas	○	○	○	○	○	○
Demasiadas vacunas administradas a la vez	○	○	○	○	○	○
La enfermedad que la vacuna evita no es peligrosa	○	○	○	○	○	○
El/la niño/a no está a riesgo para la enfermedad que la vacuna evita	○	○	○	○	○	○
Mejor pasar la enfermedad que recibir la vacuna	○	○	○	○	○	○
Mala experiencia con una vacuna en uno mismo, en los/as hijos/as o en alguien próximo	○	○	○	○	○	○

Otros motivos *(por favor especifique)*:

----

10 - ¿Cree usted que dispone de la formación y/o materiales suficientes para resolver adecuadamente las dudas sobre vacunación que le pueden plantear las familias?

☐ Sí

☐ No

11 - Señale la o las opciones de las cuales querría disponer usted de entre las que se proponen a continuación para resolver mejor las dudas sobre vacunación que le plantean las familias *(si ha respondido "sí" a la pregunta anterior, puede dejar esta pregunta en blanco o contestarla igualmente)*:

☐ Material informativo - papel

☐ Material informativo - online

☐ Sesiones formativas sobre temas específicos de vacunación

☐ Otros *(por favor especifique)*:

Muchas gracias por su colaboración. Le haremos llegar los resultados de este estudio próximamente.

Si quiere añadir algún comentario, puede hacerlo a continuación:

SURVEY FOR HEALTH PROFESSIONALES ON SYSTEMATIC CHILDHOOD VACCINATION

This survey is part of a study about systematic childhood vaccination carried out by the Public Health Agency of Barcelona directed towards health care providers in primary care centers in the city of Barcelona. Participation in this study is voluntary and anonymous and your responses will be treated confidentially. Thank you for your collaboration.

General data

You are:

☐ Female
☐ Male

Age ....years

Please indicate your profession

☐ Medicine
☐ Nursing

Indicate the approximate years that you have been working in this profession
.... years

¿Do you have children?

☐ No
☐ Yes

Questions about vaccination

1- If today you had a child, ¿which vaccines from the current systematic childhood vaccination calendar would you administer to them? Please indicate if you would administer them during the time period which is recommended by the calendar (YES), if you would administer them later (LATE), if you have doubts (DOUBTS) or if you would not administer to them (NO).

	YES	LATE	DOUBTS	NO
Diphtheria vaccine				
Tetanus vaccine				
Whooping cough vaccine				
Poliomyelitis vaccine				
H. influenzae b vaccine				
Hepatitis B vaccine				
Meningococcal C vaccine				
Hepatitis A vaccine				
Measles vaccine				
Rubella vaccine				
Mumps vaccine				
Human papilloma virus (HPV) vaccine (girls)				
Varicella vaccine				
Pneumococcal vaccine				

Which combined vaccines would you administer?

	YES	LATE	DOUBTS	NO
dTpa vaccine<br>(diphtheria, tetanus, whooping cough)				
Pentavalent vaccine<br>(dTpa, polio, H. influenzae b)				
Hexavalente vaccine<br>(dTpa, polio, H. influenzae b, hepatitis B)				
MMR vaccine<br>(measles, mumps, rubella)				

2- **What PROBABILITY do you consider that an unvaccinated child who is NOT vaccinated/immunized have of contracting the following disease? Consider a 10-year period of the current Spanish context.**

	IMPOSSIBLE	NOT VERY PROBABLE	NEITHER PROBABLE NOR IMPROBABLE	LIKELY	VERY LIKELY	DON'T KNOW/NO RESPONSE
Diphtheria						
Tetanus						
Whooping cough						
Poliomyelitis						
H. influenzae b disease						
Hepatitis B						
Meningococcal C disease						
Hepatitis A						
Measles						
Rubella						
Mumps						
Varicella						
Pneumococcal diseases						

3- **This question is about the SEVERITY of immuno-preventable disease. How serious do you consider tan an 8-year old child who is not vaccinated/immunized becomes infected with the following diseases?**

	NOT AT ALL SERIOUS	NOT VERY SERIOUS	SOMEWHAT SERIOUS	SERIOUS	VERY SERIOUS	DON'T KNOW/NO RESPONSE
Diphtheria						
Tetanus						
Whooping cough						
Poliomyelitis						
H. influenzae b disease						
Hepatitis B						
Meningococcal C disease						
Hepatitis A						
Measles						
Rubella						
Mumps						
Varicella						

Pneumococcal diseases						

4- This question is about the SAFETY of vaccines. How safe do you consider that each of the following vaccines are?

	DANGEROUS	NOT VERY SAFE	SAFE	VERY SAFE	TOTALLY SAFE	DON'T KNOW/NO RESPONSE
Diphtheria vaccine						
Tetanus vaccine						
Whooping cough vaccine						
Poliomyelitis vaccine						
H. influenzae b vaccine						
Hepatitis B vaccine						
Meningococcal C vaccine						
Hepatitis A vaccine						
Measles vaccine						
Rubella vaccine						
Mumps vaccine						
HPV vaccine (girls)						
Varicella vaccine						
Pneumococcal vaccine						

5- What PROTECTION do you think the following vaccines offer? Indicate the degree to which you consider that these vaccines prevent children from become infected with the diseases they were developed for:

	NO PROTECTION	NOT VERY PROTECTIVE	SOMEWHAT PROTECTIVE	PROTECTIVE	VERY PROTECTIVE	DON'T KNOW/ NO RESPONSE
Diphtheria vaccine						
Tetanus vaccine						
Whooping cough vaccine						
Poliomyelitis vaccine						
H. influenzae b vaccine						
Hepatitis B vaccine						
Meningococcal C vaccine						
Hepatitis A vaccine						
Measles vaccine						

Rubella vaccine						
Mumps vaccine						
HPV vaccine (girls)						
Varicella vaccine						
Pneumococcal vaccine						

6- This question is about the BENEFIT associated with the vaccination of a child for different people or groups. How much do you think the following people or groups benefit when a child receives the vaccines of the current systematic childhood vaccination calendar?

	NOT AT ALL	A BIT	INTERMEDIATE	SIGNIFICANTLY	A LOT	DON'T KNOW/ NO RESPONSE
The child benefits						
The community benefits (family, Friends, neighborhood…)						
Healthcare workers benefit						
The government/administration benefits						
Pharmaceutical companies benefit						

7- In the following, there are a set of different affirmations about childhood vaccinations. Please indicate your level of agreement for each question:

	NOT AT ALL IN AGREEMENT	LITTLE AGREEMENT	NEITHER AGREE NOR DISAGREE	AGREE	STRONGLY AGREE	DONT KNOW/NO RESPONSE
Children should only get vaccinated for serious disease						
Children receive more vaccines than they need						
I am concerned that the immune system of children may be weakened due to receiving an excessive amount of vaccines						

I am more likely to trust vaccines that have been around longer than newer ones						
Vaccines are one of the safest sanitary measures						
Thanks to scientific research, vaccines are increasingly better and effective						
Vaccines strengthen the immune system						
It is better for children to develop immunity by having the illness than through vaccination						
Continuing to vaccinate children against Polio in Spain is acceptable even though it has been eliminated from the country						
At least one vaccine in the vaccination calendar contains thiomersal						
Vaccines in the current vaccination schedule are influenced by illegitimate governmental interests						
Vaccines in the current vaccination schedule are influenced by illegitimate pharmaceutical interests						
MMR vaccine can cause autism						
The varicella vaccine can cause an attenuated varicella						
At least one vaccine in the vaccination calendar contains thimerosal						
The amount of thimerosal in vaccines can cause neurotoxicity						
At least one vaccine in the vaccination calendar contains aluminum						
The amount of aluminum in vaccines can cause neurotoxicity						
Having an egg allergy is a contraindication for MMR vaccine						

People in my immediate environment are in favor of vaccination						

8- In your professional practice, have you attended to families with doubts or against some vaccine?

☐ Yes
☐ No (Continue to question 10)

9- The following are a set of diverse motives described as important reasons for having vaccine doubts or refusal to vaccinate by families. Please consider the IMPORTANCE of the following reasons that you believe vaccine hesitant families which you attend have.

	NOT AT ALL IMPORTANT	LITTLE IMPORTANCE	SOMEWHAT IMPORTANT	SIGNIFICANTLY IMPORTANT	VERY IMPORTANT	DON'T KNOW/ NO RESPONSE
Doubts about the safety of vaccines						
Doubts about the efficacy of vaccines						
Receiving too many vaccines at the same time						
The disease which the vaccine protects against is not dangerous						
The child is not at risk of becoming infected with the disease the vaccine protects against						
Better to become ill than to receive the vaccine						
Bad experience with a vaccine in themselves, in their child, or in someone close to them						

Other reasons (please specify):

....

....

....

....

....

10- Do you believe that you have sufficient training and/or materials to solve adequately doubts about vaccines and vaccination that families may ask you?

☐ Yes
☐ No

11- Please indicate the options for which you would like information to be available to provide families with doubts. (If you responded "yes" to the previous question, you can leave this question blank or respond anyways)

☐ Information material- paper
☐ Information material – online
☐ Sessions about specific vaccine-related questions
☐ Other (please specify)

....

....

....

....

Thank you very much for your collaboration. We will make the results of the study available shortly.

If you would like to add any other comment, please do so below:

....

....

....

....

....

....

....
