# Supplementary material for: Vaccine hesitancy among paediatric nurses: Prevalence and associated factors
Source: PLoS One. 2021 May 19;16(5):e0251735. doi: 10.1371/journal.pone.0251735 (PMC8133484; doi:10.1371/journal.pone.0251735)
Supplement: Attachment — Submitted filename: Jan 2021 Vaccine Hesitancy .docx [file pone.0251735.s003.docx]

Abstract:

Line 45-46: Objective: The associated factors influencing vaccine hesitancy are multiple and complex and are not all captured by this survey. Rephrase the objective to remove this part. Is the objective a prevalence of vaccine hesitancy and views on certain vaccines?

Line 51: dichotomized into not hesitant

Abstract: Results:

Line 55: Remove A from before 32.1%.

Does sentence refer to the nurses themselves being hesitant about HPV and varicella, should state so more clearly than ‘reported’ which could mean they report patient/public hesitancy?

Line 57-59: Brackets in wrong place around the confidence intervals.

The statements supported by the Odds Ratios are perhaps too strong in their conclusion as the confidence intervals are very wide. I would recommend adjusting the strength of statement to be more suggestive, or could possibly be linked. I would not draw a strong association from these OR and CI.

The Abstract does not refer to findings where there was a large degree of agreement, such as results in lines 218-220, and instead has highlighted certain ‘controversial’ findings which I believe is misleading. Large agreement on vaccine effectiveness and safety was reported in the results which is important. A balanced view of the findings is not present in the current abstract.

Line 61: related to

Line 70: what disease is referred to here? Provide some examples of countries and immune-preventable diseases to explain.

Line 69 and 73: immune-preventable or vaccine-preventable diseases – pick one for consistent use throughout paper.

Line 82-83: rephrase the following *”association between the MMR vaccine and autism is not uncommon”*. This is a misconception and should be clearly presented as such, in its current phrasing it suggests there is a link between MMR and autism and is inaccurate.

Line 96-100: Could the authors expand here on what the key findings of this study were?

Line 102-103: as per comment on the paper objective I don’t believe this survey captures information on factors associated with VH in the study population.

Line 110: replace universe with population sample.

Line 107-111: Not enough detail provided on the study population. How did the researchers access their contact details or disseminate information? Were they registered nurses with a national nursing authority or board?

Explain cross-sectional study, should outline more information that it is a survey from the outset of methods.

Data collection Lines 114-119:

Insufficient information on the development of the questionnaire. Was the questionnaire in reference 15 validated, previously disseminated? For what population? How did the researchers decide on the relevance of all the questions, were any questions removed from the original questionnaire?

Who translated the questionnaire? How was this tested for clarity?

How were the questionnaires sent to nurses, by paper, online, by email or phone message?

Were they sent out at one point in time or on different days in the primary care centres? Explain why the survey was sent out over a 11 month period, why was this done, surely there would be different views over this length of time potentially due to media/social influences over the course of this year that can influence peoples views? A questionnaire should capture all respondents’ views at the same point in time. This is a major flaw.

Was the questionnaire collected on paper and if so who input the data into Stata, was it double checked and data cleaned?

Line 124: vaccinable diseases….see comment regarding lines 69 and 73.

Line 103 and line 125: What was the geographical area of work of the study nurses included in the study? Barcelona is one area within a larger Catalonia, were the nurses working all over Catalonia or not?

Line 130 – 135: Why do the authors talk about antigens? Would the term vaccine be more clear and consistent? What was included in the questionnaire?

Line 135: clarify exactly what responded otherwise means.

Several health behaviour theories are being mentioned in the methods e.g. line 131 Theory of Reasoned Action, Line 137 HBM, with no justification of why they are being applied.

Was the questionnaire not based on a previous questionnaire (ref 15) and did this questionnaire refer to these theories or not in it’s development?

The section on Variables is confusing and difficult to interpret without a copy of the questionnaire to outline what was on the final questionnaire? Again it is not clear how the several health theories can all be applied in one questionnaire. No explanation of the theory, relevance or what elements of the theory are used to guide the questions.

Line 154-160: what variables were used in the logistic regression model?

Line 169: provide actual number of 83% of 165, how many responses?

Line 170: remove ‘was’ from end of sentence.

Table 1: Does Vaccine hesitancy categorisation here mean they were hesitant of all vaccines or at least vaccine? Did the questionnaire capture that level of detail as suggested by lines 176-179.

Line 185: what are the 14 diseases in the schedule?

Line 190: what does these vaccines refer to? All 14 vaccines?

Line 191 and 194: provide actual numbers in brackets with 76.5%, 97.7% and 90%

Table 2: title should read that it is nurses views of these factors/ agreement with these statements.

Table 2, footnote e: was the question addressed to the parent of an 8 year old child? How were these questions phrased? Were the nurses asked their own opinion of HPV susceptibility, severity, safety and protection, not in the context of considering their own child? This is not clear.

Line 206: hesitant professionals? Nurses were the only group in the sample – replace.

Lines 222-228: was there a ‘do not know’ option answer here? Did respondents who did not enter a response here, respond to other questions in this section or not? It cannot be suggested that missing values mean the respondent did not know the answer, therefore this paragraph does not present ‘notable’ findings.

Lines 230-235:

These findings are not associated with VH – some degree of causation is suggested in the way this is written. The authors can only present facts i.e. in the vaccine hesitant groups there was a low perception of the severity of whooping cough etc.

Association is suggesting influence which cannot be determined from this study or questionnaire.

Line 234: what is meant by protecting factor?

Table 4: I would not present these factors as being associated with VH. These were views of respondents within the VH groups.

The questionnaire has not addressed the issue of herd immunity and nurses’ views of whether or not this has contributed to illness susceptibility.

Table 4: some spelling errors throughout.

Gender would typically be reported as Male and Female, rather than Man and Woman.

Lines 249-252: sentence is unclear.

Line 249 and 254: reference to Catalonia and Barcelona, see comment regarding line 103 and 125.

Line 254-257: reference to GPs and other healthcare professionals made. Can the authors refer to studies of nurses as being more relevant?

Line 262: differences in vaccination schedules between regions?

Line 264: remove ‘a decade ago’.

Line 267-268: is this not a more complex issue influenced by social media, misinformation, rather than a scarcity of efficacy studies etc.? This sentence is misleading and potentially inaccurate as it does not portray a more holistic view of all the factors influencing perceptions of HPV vaccine.

Line 268-271: Again this type of reporting is misleading and damaging if published. I cannot imagine that this was the intention of the authors, however it had a very negative suggestion.

Line 277-279: Another sentence which could be taken out of context with negative impact. Elaboration of why children who were vaccinated may still contract whooping cough is needed to present a balanced view.

Line 281: Quite a strong conclusion to make based on two questions which are not a complete reflection of the nurses’ trust in the vaccination schedule; only their views on the two questions that were asked.

Line 288-290: We cannot assume missing values mean the respondent did not know the answer.

Line 295: which result indicates less than 50% of nurses have enough knowledge on vaccination? What is defined as enough knowledge?

Line 298: this claim cannot be made. This is not clearly presented in the results as already indicated in several places in this review, association of factors influencing VH cannot be determined by this questionnaire.

Line 305-306: Is there a reference or evidence to substantiate this claim?

Line 306: First time that face to face administration of questionnaire is mentioned – lack of detail in the methods regarding data collection.

Line 308: I would determine that 2016-17 (over 11 months) is not a specific period of time but a long period of time.

Line 312: what is CAP? Expand.

Line 313-315; Again, a leading statement which is not sufficiently backed up by the method or results.

I have a concern regarding the focus on issues of vaccines mistrust and negative views in the results and discussion. The paper draws on many references for negative views of vaccinations without a balanced view of trust and positive findings from similarly conducted studies.

I also have concerns regarding the questionnaire development which draws on several health behaviour theories.

The confidence intervals and odds ratios do not appear to back up the strength of findings posed by the authors. The results do not support the conclusions sufficiently.
